# Protocols for recording morphometric measurement of Indian Pangolin (*Manis crassicaudata*)

**DOI:** 10.1016/j.mex.2020.101020

**Published:** 2020-08-04

**Authors:** Priyan Perera, Hirusha Randimal Algewatta, Hasitha Karawita

**Affiliations:** a Department of Forestry and Environmental Science, University of Sri Jayewardenepura, Sri Lanka; b IUCN SSC Pangolin Specialist Group, C/o Zoological Society of London, Regent's Park, London NW1 4RY, United Kingdom; c College of Science. Health, Engineering and Education, Environmental and Conservation Sciences, Murdoch University, South Street, Perth 6150, WA, Australia

**Keywords:** Pangolins, Scales, Body measurements, Sexual dimorphism

## Abstract

Accurate morphological description of species has essential implications in field identification and cladistics. Pangolins (Mammalia: Pholidota) are considered the world's most trafficked mammals. The Indian pangolin has a wide geographical distribution in the Indian subcontinent. However, morphoanatomical variations of *M. crassicaudata* across its range are poorly understood. The published morphoanatomical descriptions have disparities, partly due to the lack of standard protocols and procedures in morphometric data collection and reporting, thus making comparisons among different records less meaningful. This Method Article presents protocols and procedures to follow in morphometric data collection and reporting for *M. Crassicaudata.* Morphometric parameters can be measured and reported under three age classes; juvenile, sub-adult, and adult, as well as the sex to describe the species' sexual dimorphism. The proposed protocol includes 13 morphometric measurements of a pangolin body. Procedures to count and report the number of body scales with special reference to the body region of a pangolin and scale morph-type are described. Morphometry of the claws of forelimbs is described using the Curvature Linear Index [1].

## Graphical abstract

**Figure:**
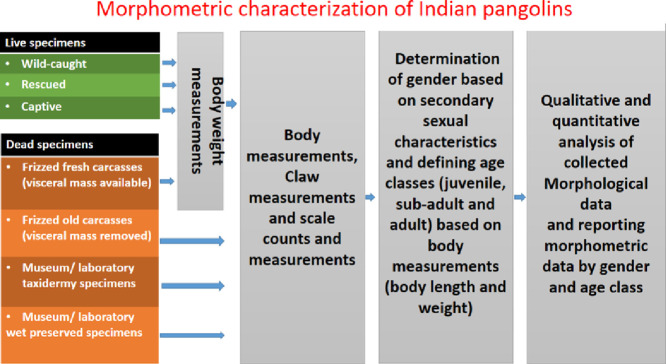

Specifications Table

**Table:** 

**Subject Area:**	Zoology
**More specific subject area:**	Animal Morphology
**Protocol name:**	Guidelines to record morphometric measurement of Indian Pangolin (*Manis crassicaudata*)
**Reagents/tools:**	Flexible measuring tape
	Vernier caliper:
	Digital weight balance:
	Digital Single Lens Reflex (DSLR) camera with 18–55 mm lens:
**Experimental design:**	Gathering morphometric measurements from Indian pangolins does not require a specific experimental design. Since the animals are rarely observed, live, dead, and museum specimens can be used for morphometric data collection. As the available information in literature are insufficient to approximate the age-size relationship, a larger sample size should be considered.
**Trial registration:**	Not applicable
**Ethics:**	This protocol involves gathering morphometric measurements from live pangolins. However, non-intrusive methods are recommended instead of standard anesthetic techniques when gathering morphometric measurements from live pangolins. Capturing and recording body measurements from live animals may require ethical clearance depending on the nature of the study. In such cases, ethical clearance should be obtained from relevant agencies. Hence, this protocol shall be applicable for ethically approved scientific research only
**Value of the Protocol:**	• Present protocol is a guide to record morphometric measurements from live, dead and museum specimens (dry and wet preserved) to describe the morphological characteristics of the Indian pangolins. • The protocol defines body regions of an Indian pangolin to aid morphometric measurement collection and guidelines to report body scale counts of an Indian pangolin • The protocol will allow easy comparison of morphometric measurements of Indian pangolins across its range

## Protocol

### Background

The Indian pangolin (*Manis crassicaudata*) is one of the four extant pangolin species in Asia and has a wide distribution in the Indian subcontinent [2], [3], [4], [5]. The Indian pangolin population in Sri Lanka is geographically isolated [6], [7], [8]. The literature suggests that there are inter-specific and intra-specific variations in morphometrics and scale frequencies among pangolin species [9]. The studies on morphological characterization of pangolin species, in general, are scarce in literature due to the difficulty of observing a sufficient number of individuals to make accurate morphological descriptions [10], [11], [12]. The Indian pangolin also shows intra-specific variations in morphometrics across its range [10,^13^,14]. However, such variations of *M. crassicaudata* within its range are poorly understood. Bodyweight, total body length (length from snout to the tip of the tail), tail length, lengths of forelimbs and hindlimbs and scale frequencies are among the commonly reported morphometric measurements [10,^12^,15].

Bodyweight measurements are essential in categorizing individuals into age classes. Periodical measurement of bodyweight is crucial in monitoring the health of captive pangolins. Most of the pangolins succumbed to death under captive conditions have shown significant weight loss before death [11]. Bodyweight and total body length are often used in defining age classes of pangolins. For instance, Irshad et al. [10] categorized Indian pangolin into three age classes: Juveniles (≤2.5 kg, 40–65 cm), sub-adults (2.51–8 kg, 66–120 cm) and adults (≥8 kg, ≥120 cm). Based on the observations of 24 specimens, Algewatta et al. [13] defined the same age classes for *Manis crassicaudata* in Sri Lanka as juvenile (≤4.3 kg, ≤56.0 cm), subadult (4.3–7.3 kg, 56–101 cm), and adult (≥7.3 kg, ≥101 cm). However, these categorizations are based on the morphometric measurement of specimens observed in respective studies. More scientific observations are required to define reliable body size ranges for different age classes.

The claw measurements can provide useful insights to the habitat conditions an Indian pangolin occupies. Wear-out f claws are likely to depend on the edaphic conditions of an individual's home range. For instance, it has been observed that the Indian pangolins living in habitats with dry and rocky habitats have more worn-out claws than those living in rainforest habitats [16], [17], [18], [19].

The scales found in different regions of the pangolins' body are different in size [3]. The smallest scales are present in the head region, and the largest ones are present in the trunk region [10]. There are intra-specific variations in total scale numbers in Indian pangolin, ranging from ~440 to 530 [3,9]. Mohapatra et al. [14] reported the scale frequency of an Indian pangolin to vary from 444 to 519 (474 ± 22). According to Ullmann et al. [9], an Indian pangolin on average bears 495.11 ± 24.19 scales. Algewatta et al. [13] reported the average scale frequency of an Indian pangolin to be 510.7 ± 20.8. The scale courts on different body regions reported by Irshad et al. [10] and Algewatta and Perera (in review) also show some deviations in numbers. These disparities in morphometric measurements can be partly attributed to different body measurements and scale counting protocols employed by the researchers. The use of different protocols in recording morphometric measurements of Indian pangolin by different researchers makes it difficult to compare records across the range of the species meaningfully. Therefore, this study proposes protocols and procedures to be followed in morphometric data collection and reporting for Indian pangolin.

### Specimens suitable to gather morphometric measurements

A wide range of specimens can be used for morphometric data gathering on Indian pangolin. These include live specimens, fresh and frozen carcasses, and museum specimens. The museum specimens may include taxidermy specimens, dry preserved complete skins, and wet preserved specimens. Although wet preservation of mammals is not common, some museums have fluid-preserved specimens of newborn and juvenile pangolins. All these different types of specimens can be used in gathering specific morphometric measurements, which are described in subsequent sections.

### Determination of sex and age category of the specimens

Although a healthy male Indian pangolin is stockily built and much larger than a female of the same age, it is difficult to distinguish males and females without examining the genital area [3,13]. The males have a visible penis, and the testicles are present within the abdominal cavity. Therefore, distinct scrotum cannot be observed in males. Females have their vulva as a pore-like structure below the anus. Both males and females have cranial pair of nipples. Well-developed mammary glands are only observable among the reproductively matured females under pregnant or lactating conditions [11]. The pangolins should be uncurled to observe the genital area and mammary glands [12].

### Handling of specimens to take physical measurements

**Fig. 1:**
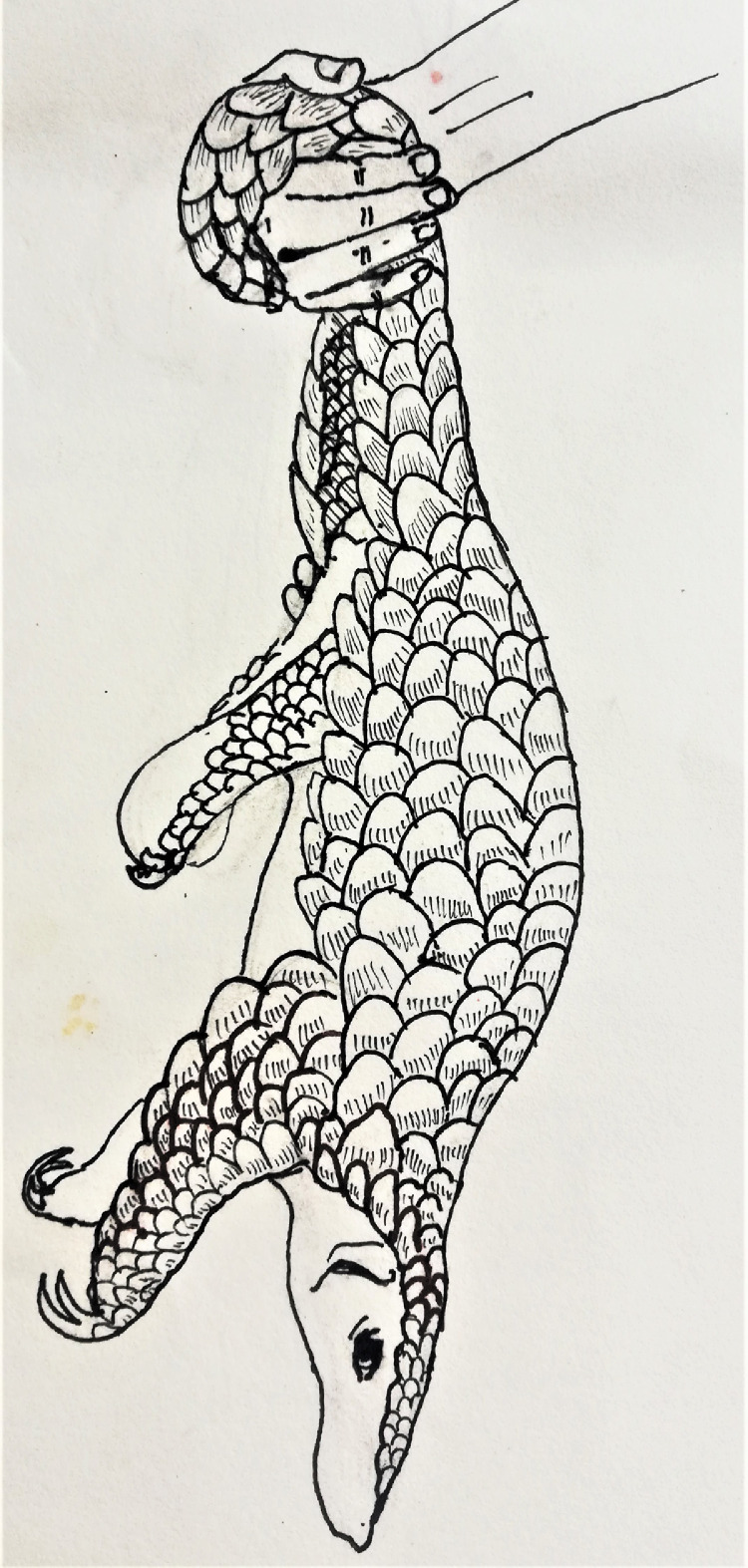
Holding a pangolin by the distal end of the tail to uncurl.

Live specimens used for morphometric studies typically include rescued or confiscated wild pangolins and pangolins in captivity in zoos. Rescued or confiscated pangolins are usually kept in cages, boxes, or gunny sacks until they are released back to wild. When taking body measurements, it should be done in a way to minimize the stress to the animal, and the measurements need to be collected as quickly as possible. Rescued or confiscated Indian pangolins usually stay tightly curled in sacks or boxes, making obtaining most body measurements extremely difficult. Hence, individuals should be uncoiled before taking body measurements. This can be done by holding the pangolin from the distal end of the tail (Fig. 1) and subjecting it to gentle up-and-down motion for a few times [12]. When handling the pangolins using hands, a pair of hard gloves should be worn to avoid damage to hands from the sharp edges of scales during the pangolin's sudden coiling around the arms. Body measurements can be taken while the animal is at an uncurled position or gently placing on a flat surface in a physiological position. Active pangolins may be caught for body measurements by firmly grasping the tail [20]. More habituated Indian pangolins in captivity can be handled much easier and can be placed in a physiological position before taking measurements.

Handling and recording body measurements from live animals may require ethical clearance depending on the nature of the study. In such cases, ethical clearance should be obtained from relevant agencies. Hence, this protocol shall be applicable for ethically approved scientific research only.

Body measurements from fresh carcasses can be obtained by placing it on a flat surface in a physiological position. Frozen carcasses should be thawed at room temperature before taking body measurements.

### Measuring bodyweight

The Indian pangolin usually stays coiled up if the threat or disturbance is persisting. Therefore, once captured, the animal can be carefully placed inside a gunny sack and then hooked into a handheld weighing scale to record the bodyweight [12]. Juvenile and sub-adult pangolins (<5 kg) may be weighed using a table spring balance. Only fresh or frozen pangolin carcasses and live pangolins shall be used to obtain bodyweight measurements.

### Taking length measurements

**Fig. 2:**
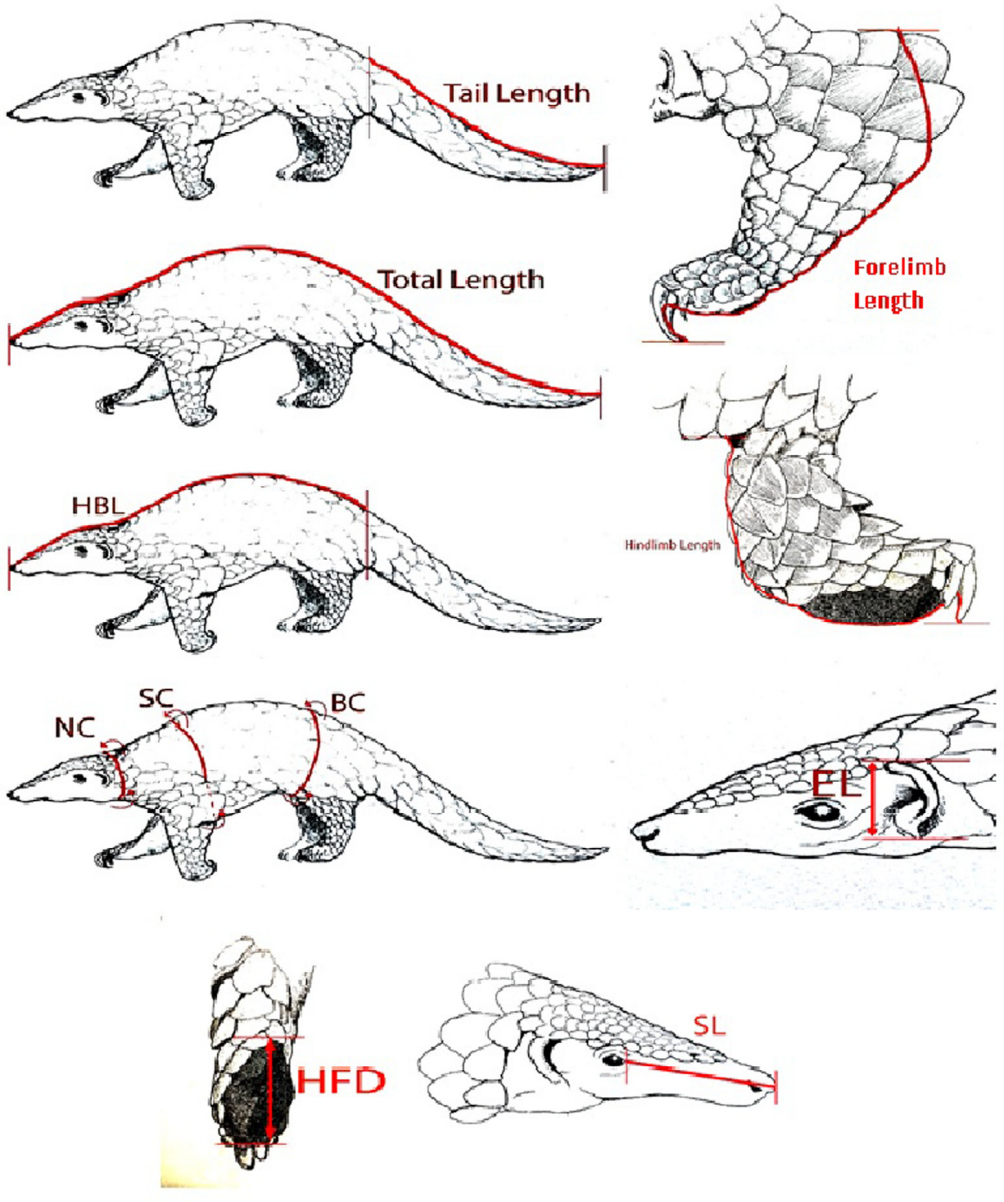
Morphometric parameters measured on Indian pangolin from Sri Lanka: HBL-physiological = Head-body length measured with the animal placed in a physiological position, Total length, TL = Tail length, Forelimb length, Hind limb length, NC = Neck circumference.

**Fig. 3:**
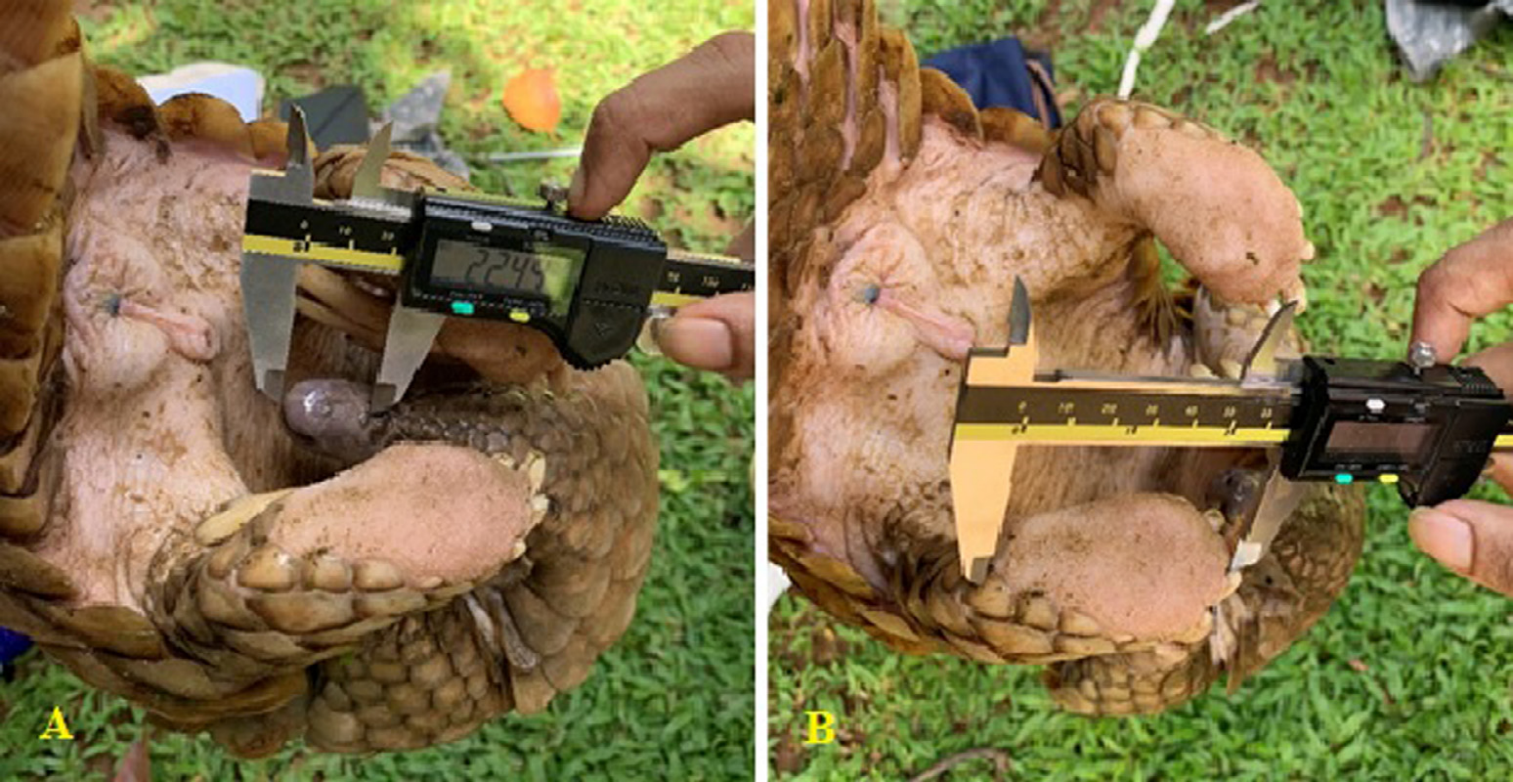
Recording (A) snout length and (B) hind foot diameter from a Vernier caliper while the animal being held by the tail.

Body length measurements are important in understanding the growth dynamics of pangolins, and they are often used in defining and classifying individuals into age classes [10]. The Indian pangolin should be in an uncurled position or in a physiological position before taking body measurements. A set of 13 different morphometric measurements is recommended to record from an Indian pangolin (Table 1). The measurement procedures and guidelines are illustrated in Fig. 1. Body length measurements are taken along the vertebral line on the dorsal surface as it is difficult to expose the ventral surface of a live pangolin. A flexible/cloth tape can be used to take all length measurements. However, a Vernier caliper is recommended to measure snout length, ear length, and hindfoot diameter (Figs. 2 and 3).

**Table 1.** Description of body measurements of an Indian pangolin.

Parameter	Description	Justification
Bodyweight (BW)	Total bodyweight	An indicator of the animal condition, growth stage and age of the animal [21]
Head length (HL)	From the upper commissure of the nostrils to the nape [22]	An indicator of the growth stage and to determine inter-specific and intra-specific variations of pangolin species [13]
Head body length (physiological) (HBL)	From the tip of the nose to the sacrococcygeal joint, following the animal's contours with the pangolin in a physiological recumbent position	An indicator of the growth, age and to characterize intra-specific variations of pangolin species [23]
Neck circumference (NC)	Measured not too tightly around the caudal portion of the neck	NC, SC and BC are useful indicators of the animal condition and growth stage/age [13,^24^,25]
Shoulder circumference / Axillary girth (SC)	Measured behind and around the shoulders [24]/ measured with a tape closely around the chest "immediately behind the forelegs" [26]	
Body circumference (BC)	Measured around the middle of stomach area.	
Tail length [27]	From the sacrococcygeal joint to the tip of the last caudal vertebral or to the last scale tip of the vertebral scale row. (upraising the tail from its base [28]	To determine the age/growth stage of an individual, and to determine inter-specific and intra-specific variations [27,23].
Hindlimb length (HLL)	Measured from the tip of the calcaneus to the distal end of the foot pad on the longest digit/ claw of the longest toe. [29,30]	To determine the age/growth stage of an individual, and to determine inter-specific and intra-specific variations [24,31]
Forelimb length (FLL)	Measurement from humeroulnar joint (elbow joint) to the end of the fore foot pad on the longest digit/ claw of the longest tow.	To determine the age/growth stage of an individual, and to determine inter-specific and intra-specific variations [23]
Total body length (TBL)	From the tip of the nose to the tip of the last caudal vertebral or to the last scale tip of the vertebral scale row	To determine the growth, and the age class of a pangolin [3,13].
Ear length (anthelix–apex) EL)	From the bottom of the fold behind the anthelix to the apex of the pinna (left and right) [29]	Useful in determining inter-specific and intra-specific variations of pangolin species [23]
Snout length (SL)	From tip of the nose to the ocular area of the head.	Useful in determining inter-specific and intra-specific variations of pangolin species [10,13]
Hind foot diameter (HFD)	Measured from the starting point of metatarsals to the distal end of the foot pad on the longest digit/ claw of the longest toe. (both feet)	As an indirect field sign, hind foot diameter can be used to predict the total body length [13,32]

Adopted from Perera et al., (in review). Weight is measured within 0.1 kg, all lengths within 0.1 cm.

### Claw measurements

**Fig. 4:**
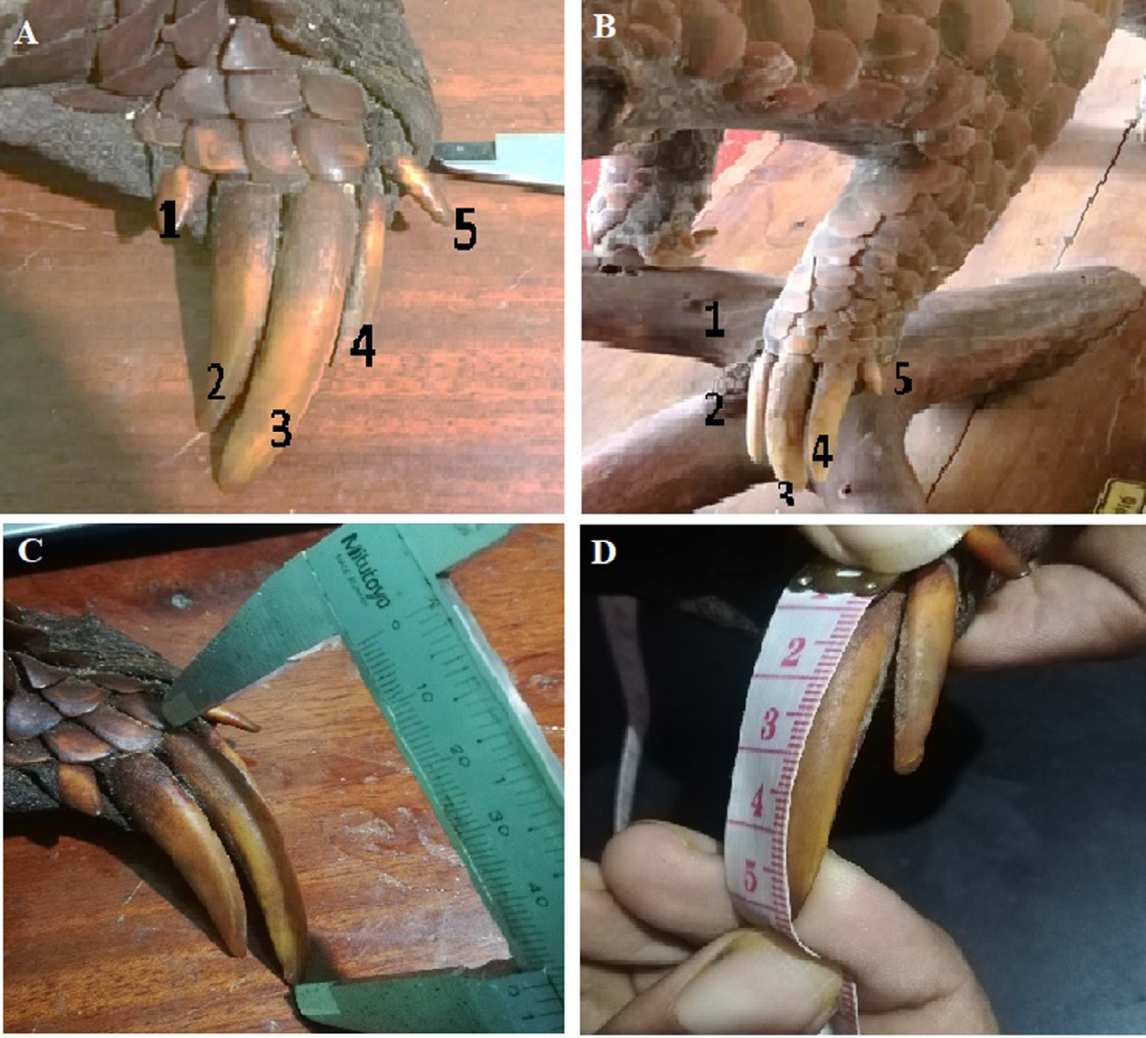
Obtaining claw measurements: A and B show the method of numbering claws. C shows the linear length measurement using a Vernier caliper. D shows measuring of curvature length [11] of a claw.

First, the claws of the forelimbs shall be numbered (1 to 5) starting from inside of the body (Fig. 4A and B). Since the first and fifth claw developments are comparatively less prominent in Indian pangolin, the measurements of middle three claws (2nd, 3rd, and 4th) shall be taken. There are two primary claw measurements. The linear length of the claws can be measured using a Vernier caliper, and the curvature length [11] can be measured using flexible/cloth measuring tape (see Fig. 4C and D) [33]. Measurements of the claws should be taken from the base of the claw to the tip of the claw along the external perimeter. These two parameters shall be used to compute the Claw Index [1] and Linear to curvature length ratio (LCR) using the following formulae [28].

$$\text{Claw}\;\text{Index}\;\left(\text{CLI}\right)=\left[\frac{\text{Curvature}\;\text{Length}\left(\text{CL}\right)-\text{LinearLength}\;\left(\text{LL}\right)}{\text{Curvature}\;\text{Length}\left(\text{CL}\right)+\text{LinearLength}\;\left(\text{LL}\right)}\right]*100$$

$$\text{Linear}\;\text{to}\;\text{curvature}\;\text{length}\;\text{ratio}\left(\text{LCR}\right)=\left[\frac{\text{LinearLength}\;\left(\text{LL}\right)}{\text{Curvature}\;\text{Length}\;\left(\text{CL}\right)}\;\right]*100$$

### Determining scale frequencies and scale arrangement

**Fig. 5:**
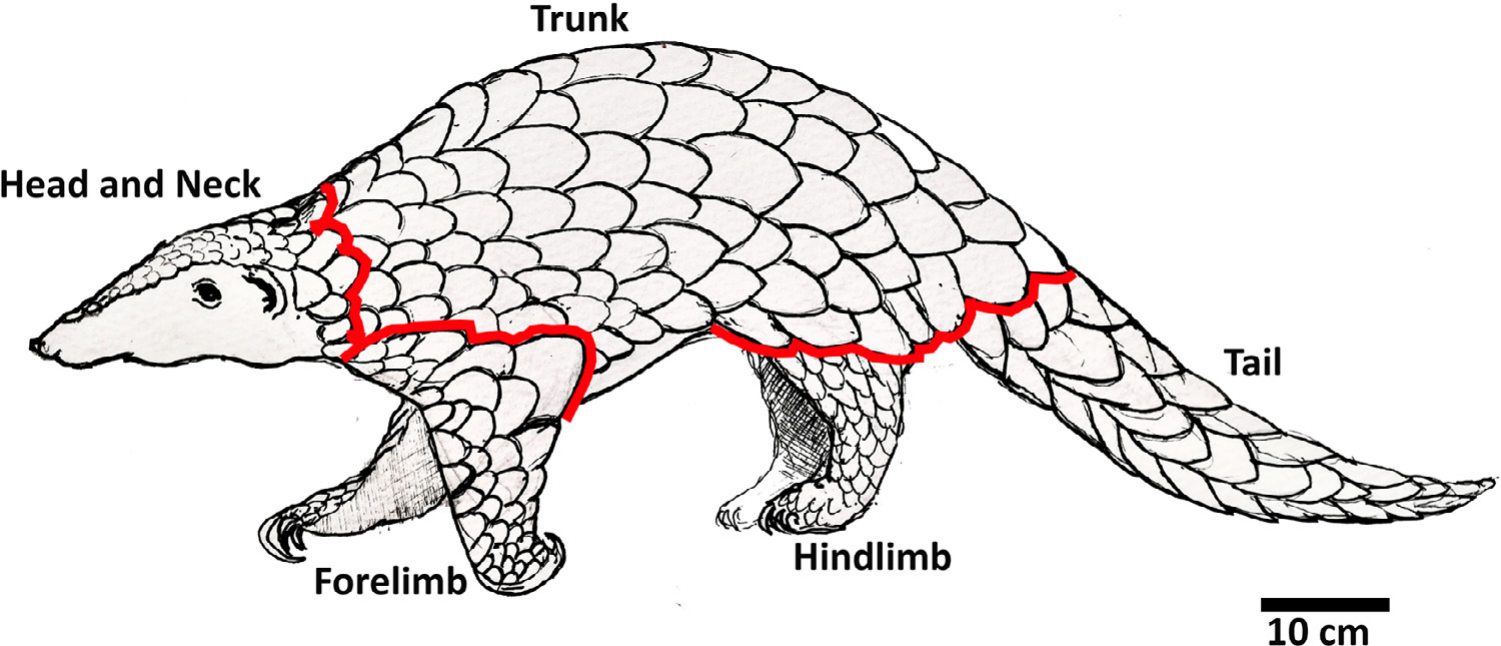
Definition and demarcation of body regions for scale counts.

**Fig. 6:**
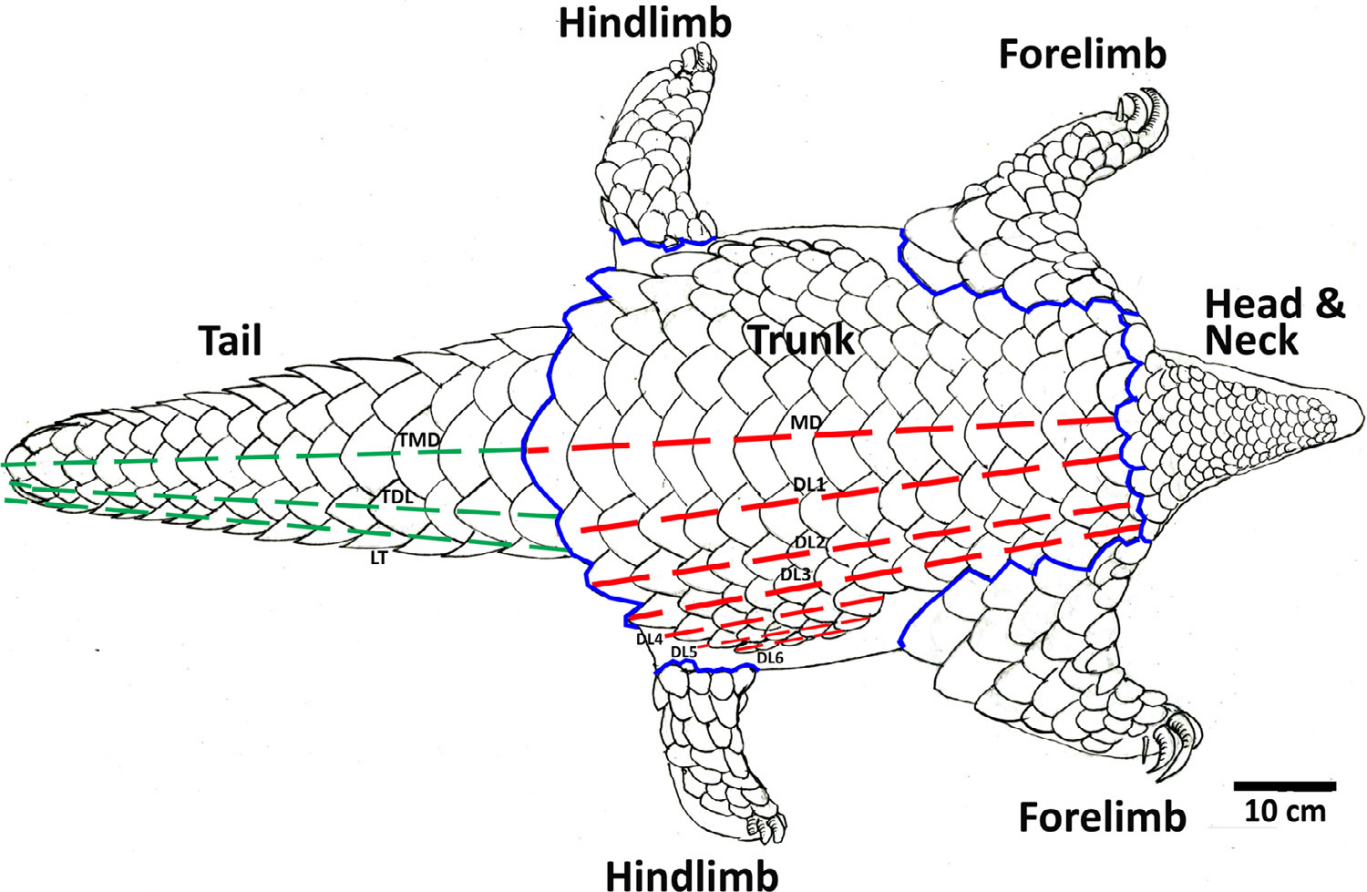
Definition and demarcation of scale rows and scale measurements. Trunk mediodorsal scale row (MD), Trunk dorsolateral scale rows (DL1 to DL6), Tail mediodorsal scale row (TMD), Tail dorsolateral scale row (TDL), Tail lateral scale row (LT).

The scales on different regions of the body of an Indian pangolin differ in frequency, size, and shape. Hence it is recommended to perform scale counts by pre-defined body regions. Five body regions are defined herein to perform and report scale counts: head, trunk, forelimbs, hindlimbs, and tail (Fig. 5). Scale counts shall be performed manually as well as using photographs. Dorsal, lateral and ventral surfaces of each specimen should be photographed, preferably using a Digital Single Lens Reflex (DSLR) camera (18–55 mm) to ensure all the scales are captured in the photographs for counting. The photographs may be analyzed using image processing software such as Adobe Photoshop (Adobe World Headquarters, San Jose, California, U.S.) and ImageJ™ (Research Services Branch, National Institute of Mental Health, Bethesda, Maryland, USA).To avoid double counting of scales, each counted scale shall be marked on the image, and counting shall be performed by body regions (Figs. 5 and 6). In cases of missing scales due to fall-off, but could be determined with confidence that a scale was once present (when there is an obvious scale bed), they shall be included in the scale count [9].

Scales in the body region of an Indian pangolin are arranged in 13 longitudinal rows while the dorsal scales of the tail region are arranged in 5 rows [23]. These rows can be identified and demarcated as illustrated in Fig. 6. It is further recommended to report scale counts in the body and tail region to facilitate comparisons and reveal inter-specific and intra-specific variations in scale frequencies and arrangements.

### Scale length, width and grooves

**Fig. 7:**
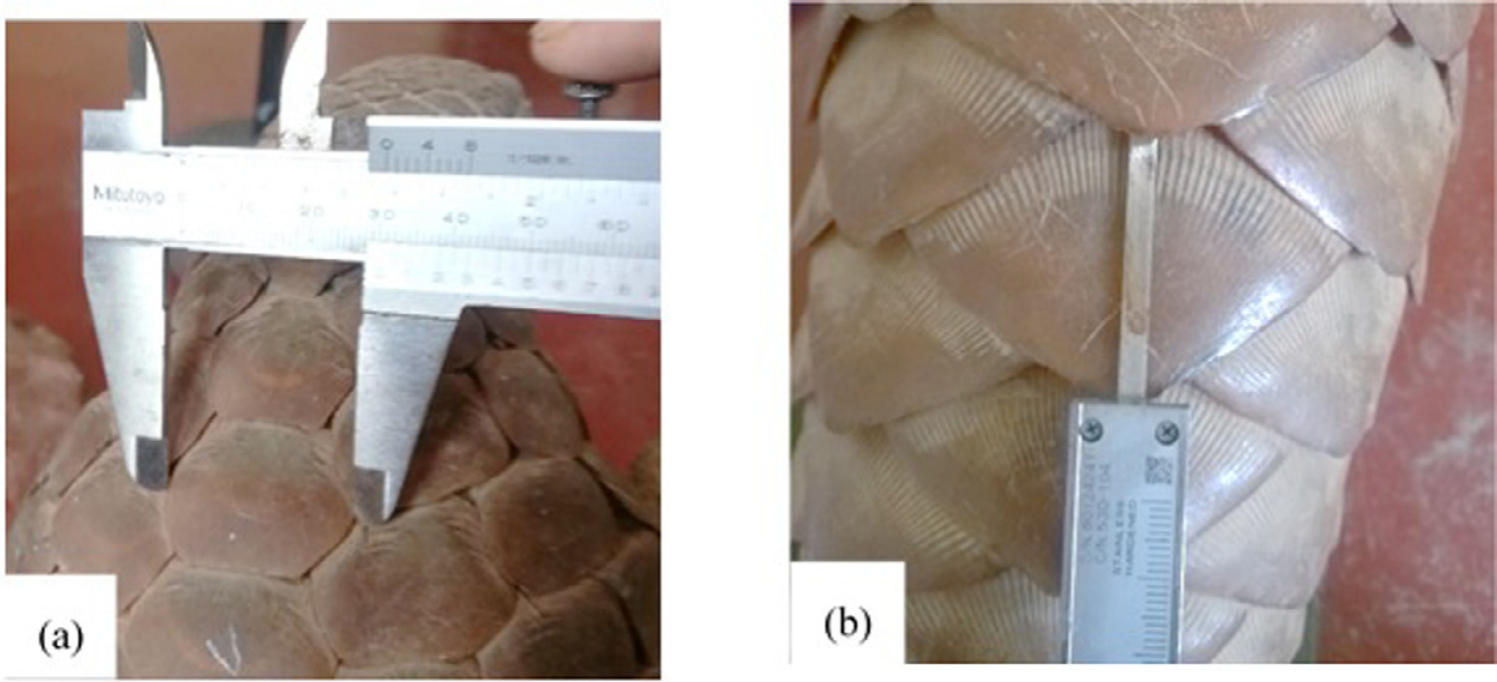
Measurement of scale (A) width and (B) length.

Length and width of a scale can be measured using a Vernier caliper (Fig. 7). The distance between the two widest points of a scale shall be considered as the width of the scale (Fig. 7A), and the distance between the base and the tip of the scale shall be considered as the length of the scale (Fig. 7B).

**Fig. 8:**
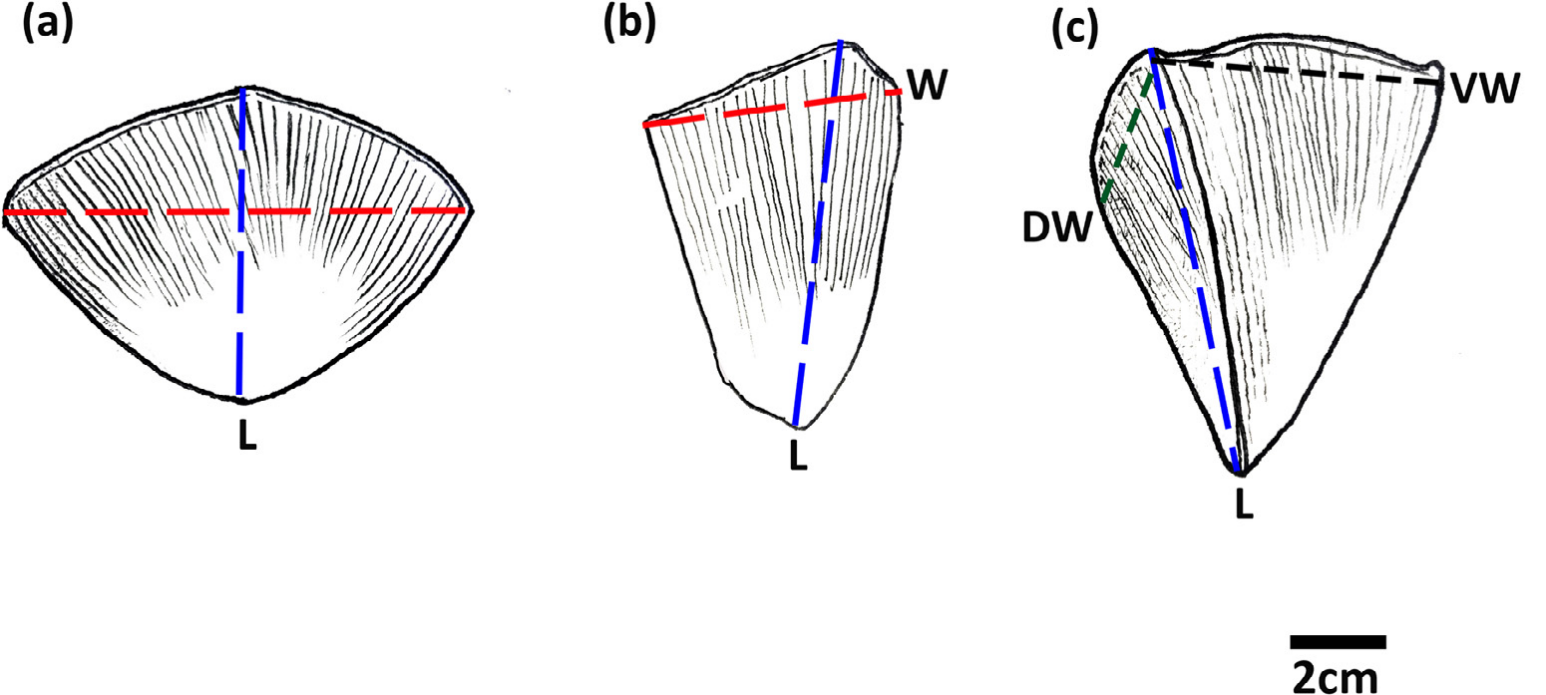
Length and width measurement guidelines for (a) broad rhombic/scapular shaped (b) elongated kite-shaped and (c) folded shaped scales; Width (W), Length (L), Dorsal width [34], Ventral width (VW).

As described by Algewatta et al. (in review), three main scale morph-types can be observed on Indian pangolin specimens. The three morph-types are broad rhombic/scapular shaped scales, elongated kite-shaped scales, and folded shaped scales. Guidance on taking length and width measurements of each scale morph-type is illustrated in Fig. 8.

**Fig. 9:**
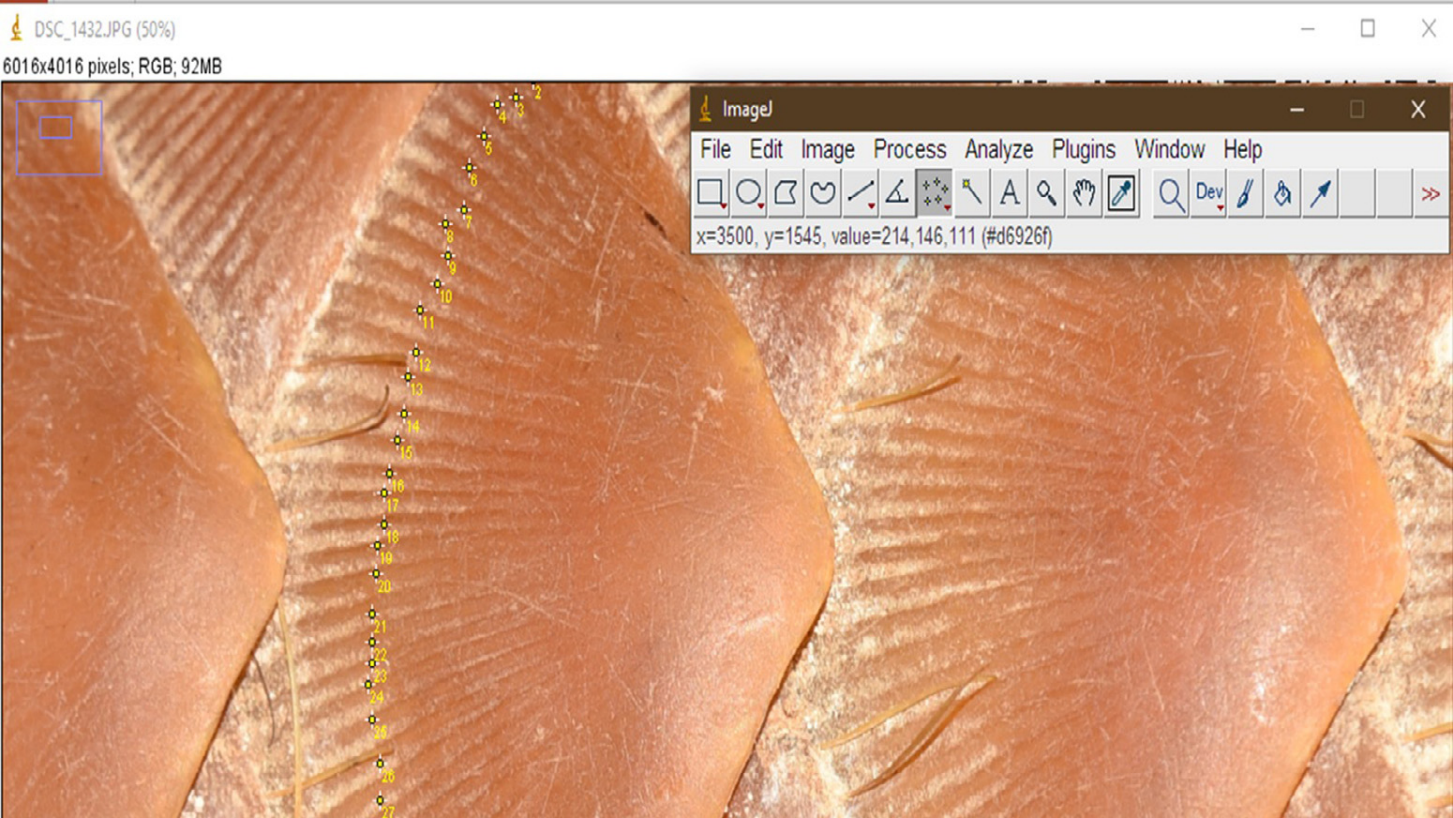
Counting scale grooves using the ImageJ™ software.

Grooves on the scales shall be counted manually or using photographs visualized in an image processing software such as ImageJ™ (Research Services Branch, National Institute of Mental Health, Bethesda, Maryland, USA) (Fig. 9). Scales with damaged or worn-out surfaces should not be considered for counting the number of scale grooves [35].

### Reporting of morphometric measurements by age classes

Morphometric parameters can be measured and reported under three age classes; juvenile, sub-adult, and adult, as well as by the sex to describe the sexual dimorphism. Although body measurement ranges have been suggested for each age category for *M. crassicaudata* in the literature [10,13], there exists a more significant morphoanatomical variation. Hence, the proper definition of age classes needs further scientific observations on the species.

## Declaration of Competing Interest

The authors declare that they have no known competing financial interests or personal relationships that could have appeared to influence the work reported in this paper.

## References

[1] Adams-Hosking C., Grantham H.S., Rhodes J.R., McAlpine C., Moss P.T. (2011). Modelling climate-change-induced shifts in the distribution of the koala. Wildl. Res..

[2] Mahmood T., Akrim F., Irshad N., Hussain R., Fatima H., Andleeb S., Aihetasham A. (2019). Distribution and illegal killing of the Endangered Indian pangolin Manis crassicaudata on the Potohar Plateau, Pakistan. Oryx.

[3] Mahmood T., Mohapatra R.K., Perera P., Irshad N., Akrim F., Andleeb S., Waseem M., Sharma S., Panda S. (2020). Indian pangolin Manis crassicaudata (Geoffroy, 1803). Pangolins.

[4] Mahmood, T., Challender, D., Khatiwada, A., Andleeb, S., Perera, P., Trageser, S., Ghose, A. & Mohapatra, R.*Manis crassicaudata* The IUCN Red List of Threatened Species 2019 [cited 2020 02 April]; Available from:10.2305/IUCN.UK.2019-3.RLTS.T12761A123583998.en.

[5] Karawita H., Perera P. (2020). A method for rapid assessment of the distribution and conservation status of Indian Pangolin (Manis crassicaudata) in an extended geographical region. MethodsX.

[6] Perera P., Karawita H. (2020). An update of distribution, habitats and conservation status of the Indian pangolin (Manis crassicaudata) in Sri Lanka. Glob. Ecol. Conserv..

[7] Perera P., Karawita K., Pabasara M. (2017). Pangolins (Manis crassicaudata) in Sri Lanka: a review of current knowledge, threats and research priorities. J. Trop. Forest. Environ..

[8] Karawita K., Perera P., Pabasara M. (2016). Indian Pangolin (Manis crassicaudata) in Yagirala Forest Reserve Ethnozoology and Implications for Conservation. Proceedings of the International Forestry and Environment Symposium.

[9] Ullmann T., Veríssimo D., Challender D.W. (2019). Evaluating the application of scale frequency to estimate the size of pangolin scale seizures. Glob. Ecol. Conserv..

[10] Irshad N., Mahmood T., Nadeem M.S. (2016). Morpho-anatomical characteristics of Indian pangolin (Manis crassicaudata) from Potohar Plateau, Pakistan. Mammalia.

[11] Nguyen V., Clark L., Phuong T. (2014). Husbandry Guidelines Sunda Pangolin (Manis Javanica).

[12] Sulaiman M.H., Azmi W.A., Hassan M., Chong J.L. (2017). Current updates on the morphological measurements of the Malayan pangolin (Manis javanica). Folia Zool. Brno.

[13] Algewatta H.R., Perera P.K.P., Karawita H.R., Dayawansa N.P., Manawadu D., Liyanage M. (2020). Novel Developments in The Morphometric Characterization of Indian Pangolin (Manis crassicaudata) in Sri Lanka. Sci. Rep.

[14] Mohapatra R.K., Panda S., Acharjyo L., Nair M., Challender D. (2015). A note on the illegal trade and use of pangolin body parts in India. Traffic Bull..

[15] Mohapatra R.K., Panda S. (2014). Husbandry, behaviour and conservation breeding of Indian pangolin. Folia Zool. Brno.

[16] Karawita H., Perera P., Gunawardane P., Dayawansa N. (2018). Habitat preference and den characterization of Indian Pangolin (Manis crassicaudata) in a tropical lowland forested landscape of southwest Sri Lanka. PLoS ONE.

[17] Pabasara M., Perera P., Dayawansa N. (2015). A preliminary investigation of the habitat selection of Indian Pangolin (manis crassicaudata) in a tropical lowland forest in south-west sri lanka. Proceedings of the International Forestry and Environment Symposium.

[18] Karawita H., Perera P., Dayawansa N., Dias S. (2020). Dietary composition and foraging habitats of the Indian Pangolin (Manis crassicaudata) in a tropical lowland forest-associated landscape in southwest Sri Lanka. Glob. Ecol. Conserv..

[19] Karawita H., Perera P., Dayawansa N. (2017). Habitat selection and burrow characterization of Indian Pangolin (Manis Crassicaudata) in a Tropical lowland rainforest habitat in South West Sri Lanka. Proceedings of the International Forestry and Environment Symposium.

[20] Heinrich S., Wittmann T.A., Prowse T.A., Ross J.V., Delean S., Shepherd C.R., Cassey P. (2016). Where did all the pangolins go? International CITES trade in pangolin species. Glob. Ecol. Conserv..

[21] Green A.J. (2001). Mass/length residuals: measures of body condition or generators of spurious results?. Ecology.

[22] Nicks B., Delfontaine B., Canart B., VANDERBRUggEN J., Vandenheede M. (2006). Caractéristiques morphologiques des juments de Trait belge. Ann. Méd. Vét..

[23] Challender D.W.S., Nash H.C., Waterman C. (2019). Pangolins: Science, Society and Conservation.

[24] Ansell W. (1965). Standardisation of field data on mammals. Afr. Zool..

[25] McCulloch J.S.G., Talbot L.M. (1965). Comparison of weight estimation methods for wild animals and domestic livestock. J. Appl. Ecol..

[26] Lundrigan B. (1996). Morphology of horns and fighting behavior in the family Bovidae. J. Mammal..

[27] Lim N.T., Ng P.K. (2008). Home range, activity cycle and natal den usage of a female Sunda pangolin Manis javanica (Mammalia: pholidota) in Singapore. Endanger. Species Res..

[28] National Zoological Gardens of South Africa (NZGoSA). *Pangolins Guidelines and Sampling Protocols For Confiscations*. 2017; Available from:https://www.tracenetwork.org/wp-content/uploads/2018/05/nzg-apwg-sampling-protocol_pangolin-confiscations_draft1.pdf.

[29] Marti I., Ryser-Degiorgis M.-.P. (2018). Morphometric characteristics of free-ranging Eurasian lynx Lynx lynx in Switzerland and their suitability for age estimation. Wildl. Biol..

[30] Mitra S. (1998). On the scales of the Scaly anteater Manis crassicaudata. J. Bombay Nat. Hist. Soc..

[31] Carroll C., Huntington P. (1988). Body condition scoring and weight estimation of horses. Equine Vet. J..

[32] Hile M.E., Hintz H.F., Erb H.N. (1997). Predicting body weight from body measurements in Asian elephants (Elephas maximus). J. Zoo Wildl. Med..

[33] Alarcos G., Madrigal-González J., Lizana M., Flechoso F. (2019). Sexual dimorphism in the claws of the European terrapin (Emys orbicularis): potential implications for the reproductive fitness of the species. Basic Appl. Herpetol..

[34] Willcox D., Nash H.C., Trageser S., Kim H.J., Hywood L., Connelly E., Ichu G.I., Nyumu J.K., Moumbolou C.L.M., Ingram D.J. (2019). Evaluating methods for detecting and monitoring pangolin populations (Pholidota: manidae). Glob. Ecol. Conserv..

[35] Liu Z., Jiao D., Weng Z., Zhang Z. (2016). Structure and mechanical behaviors of protective armored pangolin scales and effects of hydration and orientation. J. Mech. Behav. Biomed. Mater..

